# Benzimidazoles and Plants: Uptake, Transformation and Effect

**DOI:** 10.3390/toxics10030135

**Published:** 2022-03-11

**Authors:** Radka Podlipná

**Affiliations:** Institute of Experimental Botany, Czech Academy of Sciences, Rozvojová 263, CZ-16502 Prague, Czech Republic; podlipna@ueb.cas.cz

In recent years, there has been increasing concern over the environmental risks of the so called “Emerging pollutants (EPs)” that are defined as synthetic or naturally occurring chemicals that are not commonly monitored in the environment but which have the potential to enter the environment and cause adverse ecological and (or) human health effects. EPs originate from a variety of products as human pharmaceuticals, veterinary medicines, nanomaterials, personal care products, paints, coatings etc. It has been known for several decades that pharmaceuticals dispersed in the environment, even at low concentrations (ng/L), pose risk to aquatic life and human health [1].The impact of pharmaceuticals on non-target animals have been documented extensively but the information about their impact on plant organisms is still insufficient. Plants growing in the environment contaminated by pharmaceuticals are mostly capable to uptake them and biotransform into less toxic but also into more toxic metabolites via action of special drug-metabolizing enzymes or enzymes from endogenous metabolism. Pharmaceuticals in plants could cause changes in enzyme function/expression leading to changes in their physiology. Plants are able to metabolize xenobiotics, including drugs, via action of biotransformation enzymes and drug transporters. Metabolism of xenobiotics in plants is considered to occur in three phases. Oxidation, reduction or hydrolysis of drugs represent phase I of biotransformation. In this step, reactive and hydrophilic groups are inserted or uncovered in the structures of xenobiotics. The Phase I metabolites can exhibit lower, different or higher toxicity than parent compounds depending on the structure and type of reaction. In Phase II, xenobiotics or their phase I metabolites can undergo conjugation reactions with endogenous compounds, e.g., glutathione, glucose, amino acids. The vast majority of conjugation reactions lead to detoxification of xenobiotics. The transport and storage of xenobiotic metabolites in vacuoles or cell wall represent the Phase III of xenobiotic metabolism [2].

Since drug metabolism in plants can result in more toxic compounds than the parent compound, the remediation of drugs by plants does not mean an obvious solution for drug detoxification in the environment. There is a certain risk that biological activity (and toxicity) of drugs was maintained in plants and thus consumption of plants containing drugs could be toxic for invertebrates or other animals.

Anthelmintic drugs serve for treatment of infections caused by parasitic worms—helminths. Parasitic worms include flat worms (flukes and tapeworms) andround worms, i.e., nematodes. Helminthoses of livestock result in considerable morbidity and mortality, leading to substantial socioeconomic losses. Anthelmintics are administered to wide range of animals in agriculture and aquaculture and they form a large part of the animal pharmaceutical industry [3]. Although the usefulness and necessity of anthelmintics is unquestionable, their widespread use leads to environmental contamination and might have harmful effects on non-target species due to the abundant excretion of parent substance and metabolites. Generally, control strategies have focused on egg suppression regimens that involve the frequent application of anthelmintics to individuals at intervals based on egg reappearance periods after treatment. Anthelmintic drugs can be administered in a variety of ways, including orally, by injection, or skin spray. The deworming regime varies depending on the type of equipment and the species. For example, all herd can be treated at once, which leads to large but relatively rare “inputs” of high-intensity anthelmintics entering the environment, while some farmers treat animals in small groups, resulting in regular pulses at lower doses. On the other hand, the use of long-acting or sustained-release methods (long-acting injection, slow-release boluses, or reticulorumen devices) usually result in the continuous excretion of low concentrations of residual anthelmintic [4]. Therefore in recent years, there has been a growing interest in the fate and behavior of veterinary medicinal products in manure and fertilized soils.

The benzimidazoles (e.g., thiabendazole, albendazole, mebendazole, flubendazole, fenbendazole and triclabendazole) represent the largest chemical group of anthelmintics which disrupt the cytoskeleton by selectively interacting with tubulin of parasites. Benzimidazole anthelmintics are active against gastrointestinal parasites including giardia, roundworms, hookworms, whipworms, tapeworms, pinworms, paragonimiasis, strongyles, and strongyloides, They are commonly used in the treatment of sheep, cattle, horses, fish, dogs, cats, rabbits, and seals. In humans they are considered for the treatment of cancer and fungal infection [5].

The uptake and metabolism of albendazole (ABZ) was tested in the reed and harebell. The cells were able to uptake and biotransform ABZ into several metabolites. Most of the ABZ metabolites can be considered as deactivation products, but some of them remain biologically active [6,7]. Also ribwort plantain cell suspensions along with their regenerants were able to take up and metabolize ABZ. Total of 18 ABZ metabolites were identified in the ribwort. All the ABZ metabolites (with exception of ABZ-sulfoxide) formed in the ribwort can be considered as detoxication products. However, a substantial portion of the metabolites are unstable and could be easily transformed back to the parent anthelmintic. Therefore, the consumption of the ribworts with accumulated anthelmintics or their metabolites by herbivores and leaf-eating invertebrates may present a clear risk that the animals are exposed to anthelmintics. For these reasons, after treatment with anthelmintics, animals should not be grazed on pastures (especially on those with a high ecological value) until time when the drugs are no longer present in their body [8]. Keeping grazing animals in a pasture or using their excrement to fertilize fodder fields leads to the undesirable circulation of ABZ-metabolites in the environment. The active metabolite ABZ-SO passes from the excrement (of ABZ-treated animals) to fodder plants and from there to other animals. This circulation causes chronic environmental contamination and might facilitate the development of drug resistance against helminths [9]. The development of anthelmintic resistance in equine cyathostomins under intensive use of anthelmintics was proved by Raza et al. [10].

The presence of anthelmintics in several environmental compartments has already been reported. Residues of flubendazole (FLU) were found in the leachate from agricultural manure into drainage waters and in wastewater from the pharmaceutical industry, as well as in seepage water after sprinkler irrigation of a manured area [11]. FLU has also been found in river water, in fish tissue and in sediment samples [12]. Fenbendazole (FBZ) has been also found in river water [13]. Sim et al. [14] detected fenbendazole in livestock wastewater treatment plants and simultaneously detected its metabolites (fenbendazole sulfone, p-hydroxyfenbendazole, amino fenbendazole and oxfendazole).The ribwort plantain (cell culture and regenerants) is able to uptake and biotransform these drugs into several metabolites, a way which could represent their remediation. Fenbendazole in plant cells was extensively metabolized (oxidized to sulphoxide and sulphone, hydroxylated, hydrolysed, glycosylated, and acetylated). Glycosylation represents the major fenbendazole biotransformation reaction. However, most of FLU and FBZ metabolites formed in the ribwort can be decomposed back to the biologically active parent drug. Therefore, consumption of ribworts containing anthelmintic metabolites by infected livestock could support drug-resistance development in helminths. Consumption of these ribworts could represent a risk for free-living invertebrates. Moreover, in plants themselves both anthelmintics could cause oxidative stress and decreased antioxidant defense [15].

With the aim of obtaining the most complex information, the biotransformation of fenbendazole in *Arabidopsis thaliana*, known model plant, was also studied not only as a parent drug but also to seek information regarding how its metabolites could have an impact on plant cells. The presence of fenbendazole and its metabolites (12 were identified) in *A. thaliana* influenced both gene expression and the expression of proteins. Gene expression was more affected by fenbendazole in the rosettes than in roots, protein expression more in the roots at shorter treatment (24 h) and in the leaf rosette leaves at longer treatment (72 h). Taken together, fenbendazole could affect many physiological and metabolic processes in plants [16]. FBZ was easily uptaken also by soybeans by roots and translocated it to the leaves, pods, and beans. FBZ was metabolized to 16 metabolites. In beans both, FBZ and one metabolite, were found. FBZ exposure did not affect the plant fitness or yield, but reduced activities of some antioxidant enzymes and the production of polyphenols, which represent pharmaceutically important compounds [17].

## Data Availability

Data sharing not applicable. No new data were created or analyzed in this study. Data sharing is not applicable to this article.
